# Thyme Oil Enhances the Inactivation of *Salmonella enterica* on Raw Chicken Breast Meat During Marination in Lemon Juice With Added *Yucca schidigera* Extract

**DOI:** 10.3389/fnut.2020.619023

**Published:** 2021-02-11

**Authors:** Samuel Kiprotich, Aubrey Mendonça, James Dickson, Angela Shaw, Emalie Thomas-Popo, Shecoya White, Rkia Moutiq, Salam A. Ibrahim

**Affiliations:** ^1^Department of Food Science and Human Nutrition, Iowa State University, Ames, IA, United States; ^2^Interdepartment Microbiology Graduate Program, Iowa State University, Ames, IA, United States; ^3^Department of Animal Science, Iowa State University, Ames, IA, United States; ^4^Department of Food Science, Nutrition and Health Promotion, Mississippi State University, Starkville, MS, United States; ^5^Food Microbiology and Biotechnology Laboratory, Food and Nutritional Sciences Program, College of Agriculture and Environmental Sciences, North Carolina A & T State University, Greensboro, NC, United States

**Keywords:** *Salmonella*, thyme oil, yucca extract, lemon juice, marinade

## Abstract

Enteric pathogens such as *Salmonella enterica* can survive in low pH conditions and pose a food safety threat during marinating of raw poultry meat. A study was conducted to investigate the effectiveness of thyme oil for killing *S. enterica* on raw chicken during marination in lemon juice containing yucca extract. Samples of raw chicken breast were inoculated with a five-serovar mixture of *S. enterica* (~10^8^ CFU/mL) and immersed for 2, 4, 6, and 8 h in four lemon-based marinades at 22°C: lemon juice alone (L), L with added 0.5% yucca extract (L + Y), L + Y and 0.5% thyme oil (L + Y + 0.5% TO) and L + Y + 1.0% TO. The L and L + Y served as controls. Survivors were determined by surface plating chicken homogenates on xylose-lysine tergitol-4 (XLT4) agar and XLT4 agar overlaid with non-selective agar (TAL) and counting bacterial colonies after 48 h of incubation (35°C). Marinades containing Y and TO significantly reduced initial viable populations of *S. enterica* compared to control (L and L + Y) solutions (*P* < 0.05). Based on *S. enterica* survivors on TAL medium, the L and L + Y reduced initial populations by 1.12 and 1.42 Log CFU/sample, respectively, after 8 h whereas, Log reductions caused by L + Y + 0.5% TO and L + Y + 1.0% TO, respectively, were 2.62 and 3.91 (*P* < 0.05). Numbers of survivors were higher on TAL compared to XLT4 agar (*P* < 0.05); however, the extent of sub-lethal injury caused by the marinades was not statistically significant (*P* > 0.05). The death rate of *S. enterica* increased significantly (*P* < 0.05) in the marinades containing TO (0.5 or 1.0%) compared to control (L + Y). Based on these results, thyme oil has good potential to increase the antimicrobial efficacy of lemon juice marinade against *Salmonella* on raw chicken breast and enhance the microbial safety of this popular poultry product.

## Introduction

Non-typhoidal *Salmonella enterica* are commonly implicated in foodborne disease outbreaks and are a leading cause of bacterial foodborne illnesses worldwide (1, 2). From 1998 to 2017 there were 298 salmonellosis outbreaks involving contaminated chicken meat in the United States. Those outbreaks were linked to 7,881 reported cases, 905 hospitalizations, and 4 deaths (3). *Salmonella* frequently inhabits the intestinal tract of poultry (4); therefore, a major cause of *Salmonella* contamination during poultry processing is spillage of intestinal contents during evisceration. Eradication of *Salmonella* is difficult because of numerous animal reservoirs for this pathogen and its ubiquity in the natural environment (5). Moreover, the ability of *Salmonella* to survive in poultry processing facilities increases the incidence of cross contamination to previously non-infected carcasses (6). The relatively high prevalence of *Salmonella* in retail poultry (7–9) suggests that, to date, poultry processors have been unable to completely prevent dispersion of this pathogen during production and marketing of raw poultry meat (10). Thus, due to numerous opportunities for microbial contamination in poultry processing, multiple pathogen control strategies and intervention kill steps are necessary to ensure microbial safety of poultry meat from farm to consumer (11). In this regard, antimicrobial marinade formulations may have enhanced potential as an intervention strategy to reduce enteric pathogens on raw poultry meat in the farm-to-consumer continuum.

Marination typically involves the soaking or pre-incubation of raw meats in an emulsion or water-based solution that might contain a wide variety of ingredients such as vinegar, wine, fruit juices, organic acids, spices and different aroma additives (12–15). The main purpose of marination is to improve meat tenderness, juiciness, yield, flavor, texture (14, 16, 17), and microbial quality (18). Smith and Acton (19) estimated that more than 50% of raw poultry may be marinated prior to consumption; therefore, marination presents an ideal opportunity to exploit the antimicrobial activity of certain aromatic components of herbs and spices against meat-borne pathogens. Low pH marinades containing lemon juice or vinegar have exhibited antimicrobial properties (20, 21); however, the ineffectiveness of acidic marinades with regard to completely inactivating pathogens in raw meat continues to be problematic (22). The antimicrobial activity of acidic marinades can be improved by compounds from different spices and herbs that are utilized for flavoring purposes (17, 23) as well as the addition of certain plant essential oils (EOs) (24) or EO components (25).

The EOs are extracts of aromatic plants that exhibit potent antimicrobial activity (26–29). The oily (hydrophobic) characteristic of EOs is a major impediment to their application in water-based (hydrophilic) marinades that may consist of water, salt and phosphate or mainly citrus juices such as lime or lemon juice. The EOs are not miscible in water and therefore require the addition of a surfactant for their solubilization (29–31). Considering the increasing consumer demand for more natural alternatives to synthetic food additives, natural surfactants such as yucca extract are gaining much attention from food processors. For example, yucca extract from the *Yucca schidigera* plant has FDA GRAS status and is approved for use as an ingredient in foods and beverages (Code of Federal Regulations 21CFR 172-510, FEMA number 2973).

While there is a growing body of knowledge on the application of EOs as antimicrobials in various food products, published reports on the addition of thyme oil to low pH marinades for pathogen control in poultry meat are scarce. In addition, there are no published reports on the use of yucca extract to disperse EOs in marinade solutions. Accordingly, the main objective of the present study was to evaluate the effectiveness of thyme oil for killing *S. enterica* on artificially inoculated raw chicken breast meat during marination in lemon juice with added yucca extract. A secondary objective was to determine the extent of sub-lethal injury to *S. enterica* survivors on marinated chicken breast meat.

## Materials and Methods

### Bacterial Strain and Culture Conditions

Five serotypes of *Salmonella enterica* (Enteritidis ATCC13076, Heidelberg ATCC 8326, Typhimurium ATCC 14802, Gaminara ATCC 8324, and Oranienburg ATCC 9239) were obtained from the culture collection of the Microbial Food Safety Laboratory at Iowa State University. The cultures were maintained frozen (−80°C) in brain heart infusion (BHI) broth (Difco; Becton, Dickinson and Company, Sparks, MD) with 10% (v/v) added glycerol. Each frozen stock culture was thawed under cold running water and activated in tryptic soy broth (Difco; Becton, Dickinson and Company, Sparks, MD) supplemented with 0.6% (w/v) yeast extract (TSBYE) at 35°C. Prior to each experiment, two consecutive 24-h transfers of each activated stock culture were performed in TSBYE (35°C) to prepare working cultures.

### Preparation of Inoculum

Equal volumes (6-mL) of each of the five working cultures of *S. enterica* were combined in a sterile centrifuge tube. The cells were harvested by centrifugation (10,000 × g, 10 min, 4°C) using a Sorvall Super T21 centrifuge (American Laboratory Trading, Inc., East Lyme, CT). The pelleted cells were suspended in 3.0 mL of 0.85% (w/v) NaCl (saline) to obtain a final viable cell concentration of 10 log_10_ colony-forming units (CFU)/mL for use in the *in-vitro* experiments. For inoculation of chicken breast meat samples, pelleted cells re-suspended in 30 mL of saline to give 9.0 Log_10_ CFU/mL were used. Colony counts of the cell suspensions were evaluated by serially diluting (10-fold) and surface plating samples on tryptic soy agar (Difco; Becton Dickinson) supplemented with 0.6% yeast extract (TSAYE) followed by the counting of bacterial colonies on TSAYE after incubation (35°C) for 24 h.

### Minimum Inhibitory Concentration and Minimum Bactericidal Concentration

The minimum inhibitory concentration (MIC) and minimum bactericidal concentration (MBC) of thyme oil for *S. enterica* were evaluated using a broth dilution assay (32). One milliter of filter-sterilized thyme oil was added to 99 mL of sterile BHI broth (pH 7.4) containing 0.5% (v/v) yucca extract to give an initial concentration of 1.0% (v/v). Two-fold dilutions of thyme oil (1.0%) were prepared in BHI broth with added yucca extract to obtain the following thyme oil concentrations: 0.5, 0.25, 0.125, 0.062, 0.031, and 0.015% (v/v). The *S. enterica* cell suspension (9.0 Log_10_ CFU/mL) in saline was diluted in fresh saline to obtain 7.0 Log CFU/mL. Aliquots (0.1-mL) of the diluted cell suspension were used to inoculate tubes of sterile BHI broth (10 mL/tube) containing various concentrations of thyme oil to obtain an initial *S. enterica* concentration of ~5.0 Log_10_ CFU/mL. Inoculated and non-inoculated BHI broth with yucca extract served as positive and negative control, respectively. All tubes of BHI broth were incubated at 35°C and checked for turbidity after 24 h. The MIC of thyme oil was determined as the lowest concentration at which no turbidity was observed after 24 h. To determine the MBC, 0.1 mL aliquots from the tubes showing no turbidity were surface plated on TSAYE. The TSAYE plates were then incubated (35°C) and bacterial colonies were counted after 48 h. The lowest concentration of thyme oil that demonstrated ≥99.9% (3-log) kill of the pathogen was deemed as the MBC (33).

### Preparation of Lemon Juice Marinade

Whole Sunkist^®^ lemons (Citrus *limon* L) from the same production lot were purchased from a local grocery store in Ames, Iowa. The lemons were washed with potable water and cut into halves using a sanitized knife. The lemon juice for the marinade solutions was prepared by juicing the lemons using a manual citrus juicer. The fresh juice was titrated with NaOH solution and its citric acid content was expressed as gram citric acid per 100 mL based on a citric acid standard curve. Prior to each experiment four treatment solutions including control lemon juice alone (control) were prepared by aseptically transferring 100 mL of lemon juice into each of four sterile screw-capped glass bottles. Appropriate amounts of filter-sterilized stock solutions of yucca extract (Garuda International, Inc, Exeter, CA) (34) alone or in emulsion with certified food grade thyme oil (Sigma-Aldrich, Milwaukee, WI) in lemon juice were added to the three remaining bottles of lemon juice to obtain thyme oil concentrations of 0, 0.1, and 0.2%. Except for lemon juice alone, all other marinade solutions contained 0.5% (w/v) yucca extract. The capped bottles containing the marinade solutions were vigorously shaken and held at 4°C until used.

### Inoculation of Lemon Juice Marinade Solutions

For *in vitro* time-kill studies, 20-mL aliquots of each of the four marinade solutions were aseptically transferred to separate sterile 50-mL plastic tubes and inoculated with 0.2 mL of a five-serovar concentrated cell suspension (10 Log_10_ CFU/mL) of *S. enterica* to obtain a final viable cell concentration of 8.0 Log_10_ CFU)/mL. Each inoculated treatment solution was thoroughly mixed by vortexing and samples were removed for microbial analysis at 0, 3, 6, 9, 12, and 15 min. Time 0 min represented about 15 s of exposure of the pathogen to the marinade before the samples were transferred to buffered peptone water (BPW; Difco) for further dilution and plating.

### Preparation and Inoculation of Chicken Breast Meat

Fresh, skinless chicken breast filets were obtained from a local grocery store and transported on ice in a cooler to the Microbial Food Safety Laboratory at Iowa State University. The filets were refrigerated at 4°C and used within 24 h of purchase. Five chicken breast filets were randomly selected, and samples (~50 g each) of chicken breast meat were aseptically excised using a sanitized cylindrical plastic corer 30 mm in diameter. Each sample was inoculated with 0.1 mL of *Salmonella* (~9.0 log CFU/mL) in order to obtain a final concentration of ~8.0 Log_10_CFU/sample. The inoculum was spread over the surface of the meat sample using a sterile bent glass rod. The inoculated chicken breast slices were then held for 30 min at ambient temperature (22 ± 1°C) in a laminar flow bio-hazard chamber (with the blower on) and for an additional 1.5 h without the blower to allow for cell attachment to the meat surface and drying of the inoculum.

### Marination of Inoculated Chicken Breast Meat

For marination, four 50-g samples of inoculated chicken breast slices were each transferred to a separate sterile beaker containing a marinade treatment solution at 22 ± 1°C. The four marinade solutions consisted of lemon juice only, and lemon juice with 0, 0.5, and 1.0% (v/v) thyme oil. Each of the three latter marinades contained 0.5% (w/v) yucca extract. Chicken breast meat samples were immersed (inoculated side down) in the marinade solutions with a marinade to meat ratio of 2:1 (100 mL/50 g). Meat samples were removed from the marinade and drained for 30 s on a sanitized stainless-steel grill before being analyzed for *Salmonella* survivors.

### Microbiological Analysis

Samples of non-inoculated raw juice were analyzed for aerobic plate count (APC) and naturally occurring salmonellae. In this respect, one-mL samples of juice were each added to 2-mL of double-strength (2X) buffered peptone water (BPW; pH 7.2). To determine the APC, aliquots (1.0- and 0.1-mL) of the diluted juice were surface plated on TSAYE followed by incubation (35°C) and counting bacterial colonies after 48 h. For enumerating naturally occurring salmonellae, aliquots of the diluted juice were surface plated on XLT agar overlaid with TSAYE (TAL) followed by incubation (35°C) for 48 h. For the *in-vitro* study, microbiological analysis of the inoculated lemon juice was performed to determine *Salmonella* survivors after 3, 6, 9, 12, and 15 min of inoculation. Ten-fold serial dilutions of each treatment solution were prepared in BPW. Aliquots (0.1-mL) of appropriate dilutions were surface plated (in duplicate) on XLT-4 agar and XLT-4 agar overlaid with TSAYE (TAL). The inoculated agar plates were incubated at 35°C, and bacterial colonies were counted after 48 h. *Salmonella* survivors on chicken breast meat were determined after 2, 4, 6, and 8 h of marination. At each sampling time, the meat samples were drained for 30 s on a sanitized stainless steel grill then transferred to Seward Stomacher sterile strainer/filter bags (Fisher Scientific, Fair Lawn, NJ) each containing 50 mL of 2X BPW. The bagged samples were each pummeled for 1.0 min in a laboratory stomacher blender operating at medium speed. Aliquots (1-mL) of the sample homogenate were serially diluted in BPW and 0.1-mL portions were spread-plated on both XLT-4 agar and TAL media. The inoculated agar plates were incubated (35°C) and bacterial colonies were counted after 48 h.

### Calculation of *D*-Values

The *D*-values (time of exposure to marinade that results in 90% reduction in viable salmonellae) were determined by plotting the log number of survivors per ml of marinade or per sample (marinated chicken) vs. exposure time using Microsoft Excel 2000 Software (Microsoft Inc., Redmond, WA). Using linear regression analysis the line of best fit for each set of data was determined. The D-value was evaluated by calculating the negative reciprocal of the slope of the regression line.

### Determination of Sub-lethal Injury

The TAL medium was used to recover both non-injured and sub-lethally injured *Salmonella* and was prepared by aseptically layering 14 mL of sterile TSAYE (49°C) onto 20 mL of solidified XLT-4 agar in petri dishes (35). Plates of solidified TAL media were used within 2 h after preparation for surface-plating samples. Sub-lethal injury in the surviving *Salmonella* population was determined as described by Wuytack et al. (36). Briefly, the numbers bacterial colonies recovered on XLT4 agar and TAL media were used to calculate the reduction factor (RF) after each exposure of the pathogen to the marinade. The RF is the ratio of *Salmonella* colony counts (CFU/ml) of the control to that of the treated sample. For each sampling time, the log of the RF was calculated for *Salmonella* recovered on each of the two plating media using the following equation:

$$\begin{array}{l} Log\;RF=Log\left(\frac{CFU\;before\;treatment}{CFU\;after\;treatment}\right) \end{array}$$

For each sampling time, the log RF values for XLT4 agar and the TAL medium were plotted on the Y-axis and X-axis, respectively. Linear regression lines were fitted through the data points and sub-lethal injury was determined when the values of the slope and intercept deviated significantly (*P* < 0.05) from 1.0 and 0, respectively (36, 37).

### Measurement of pH of Marinades

The pH value of each marinade was measured initially (0 h) and after 2, 4, 6, and 8 h of immersion of non-inoculated raw chicken breast meat in the marinades. The pH measurements were performed using an Orion Model 525 pH meter (Orion Research, Inc., Boston, MA) fitted with a glass electrode.

### GC-MS Analysis of Thyme Oil

The thyme oil was analyzed with Agilent Technologies Model 6890A Gas Chromatography system coupled to a Model 5973N inert XLMSD with Triple–Axis Detector. An Agilent Rxi-5SilMS (30 m × 0.25 mm × 0.25 mm) Capillary column was used, and each injected sample consisted of 1 μL of essential oil diluted in 1.0 ml Hexane (HPLC grade) using split-less injection. The inlet temperature and the helium flow rate were 250°C and 1.0 mL/min, respectively. Ionization voltage was 70 eV with interface temperature of 280°C. The MS source temperature was 230°C and the MS Quad temperature was 150°C with the temperature sequence was as follows: initial temperature 50°C, ramp 5°C per min to 180°C, then 10°C per min to 280°C with a total run time of 36 min per sample. A mixture of homologous series of normal alkanes from C10 to C26 was analyzed under the same conditions as listed above. The compounds present in the essential oil were identified by comparing the mass spectra of each component with those from National Institute of Standards Technology (NIST) by Automated Mass Spectral Deconvolution and Identification System (AMDIS). The identification was also based on a comparison between the literature and estimated Kovat′s retention indices using the formula:

RI_*x*_ = 100 [*n* + (*t*_*x*_ – *t*_*n*_)/(*t*_*n*__+1_ – *t*_*n*_)] ((38)). The *t*_*n*_ and *t*_*n*__+1_ represent retention times of the reference normal alkane hydrocarbons eluting closely before and after the chemical compound to identify “*x*.” The *t*_*x*_ is the retention time of that compound “*x*” whereas “*n*” represents the number of carbons.

### Statistical Analysis

All experiments were performed in triplicate and the results were reported as averages. The SAS software (SAS version 9.3, SAS Institute, Cary, NC) was used for two-way Analysis of Variance (ANOVA) to evaluate treatment means with significant differences. The Welch test was used to determine significant differences between paired treatments. Mean pH values were analyzed by using JMP Pro statistical software version 15 (SAS Institute, Inc., Cary, NC). The pH means were evaluated for significant differences at a 5% significance level using the Student's *t*-test.

## Results

### Minimum Inhibitory Concentration and Minimum Bactericidal Concentration

Of the thyme oil concentrations ranging from 0.015 to 1.0% (v/v) in BHI broth (pH 7.4), 0.03 to 1.0% thyme oil inhibited growth of *S. enterica*. No turbidity was observed in the respective tubes of broth. The MIC of thyme oil for the pathogen in BHI broth (35°C) for 24 h was 0.03%. The MBC of thyme oil was 0.06%, which resulted in 3.2 Log CFU/mL reduction in initial viable count of *S. enterica*.

### Viability of Pathogens in Marinade Solutions

**Figure 1:**
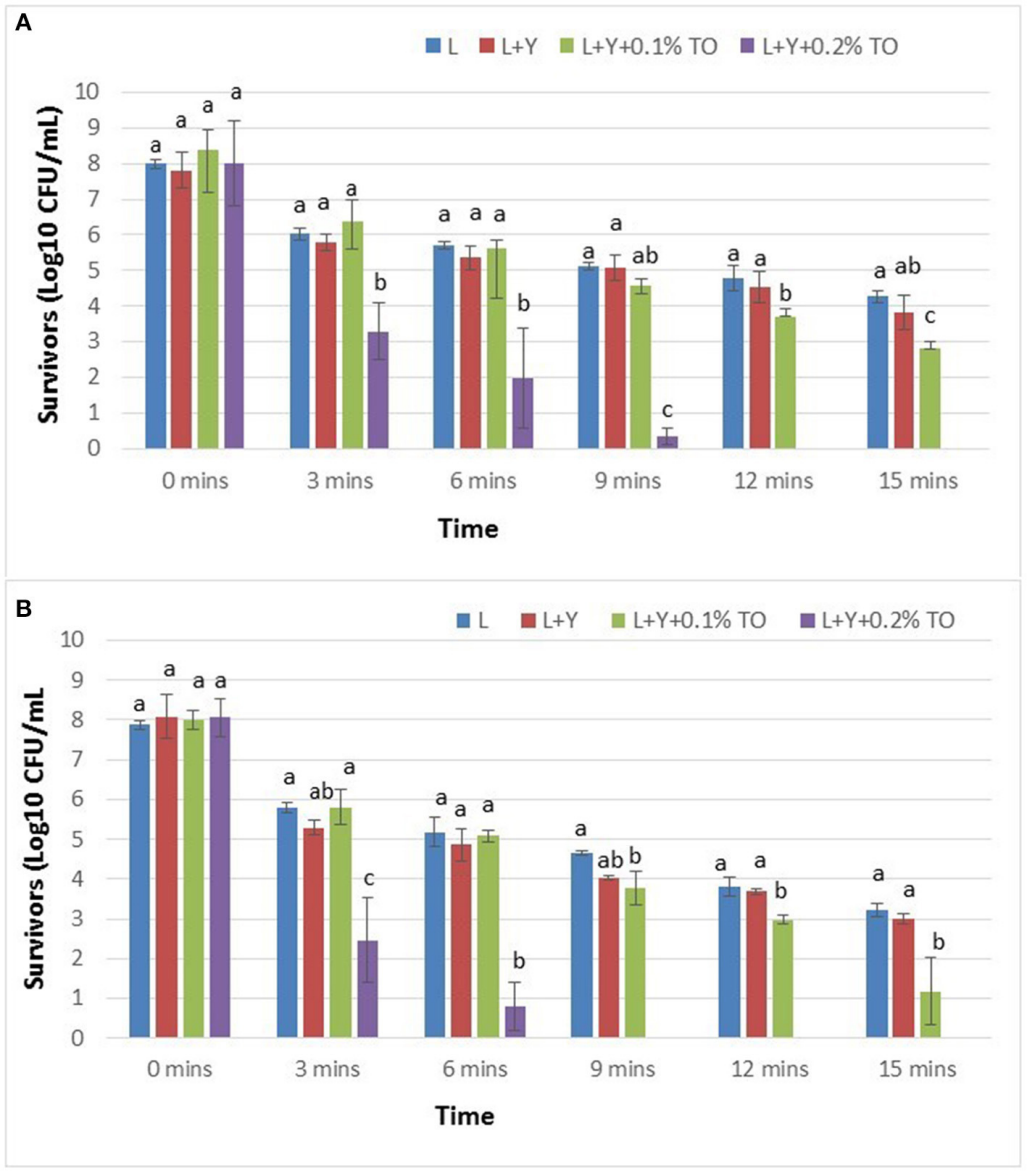
Survivors of *Salmonella enterica* planktonic cells in lemon juice marinades based on bacterial colony counts on TAL medium **(A)** and on XLT4 agar **(B)**.

The APC and numbers of viable salmonellae in non-inoculated raw lemon juice were each <3.0 CFU/mL. Figures 1A,B show numbers of *S. enterica* survivors in artificially inoculated lemon juice marinade solutions based on bacterial colony counts on TAL medium (Figure 1A) and XLT-4 agar (Figure 1B). The average initial viable count of *S. enterica* in marinade solutions was 8.0 ± 0.4 Log_10_ CFU/mL. Lemon juice alone decreased numbers of the pathogen from ~8.0 to 4.3 Log CFU/mL after 15 min (Figure 1A). Survivors in lemon juice with only yucca extract were consistently lower than those in lemon juice alone; however, differences were not statistically significant (*p* > 0.05) (Figure 1A). In lemon juice containing yucca extract and thyme oil (0.1%), initial numbers of *S. enterica* decreased from ~8.0 log CFU/mL (0 min) to 6.38, 5.62, 4.58, 3.70, and 2.81 Log CFU/mL after 3, 6, 9, 12, and 15 min, respectively (Figure 1A). Lemon juice with thyme oil (0.2%) exhibited the highest antibacterial effect whereby initial numbers of the pathogen decreased from ~8.0 (0 min) to 3.29, 1.96 and <1.0 Log CFU/mL, respectively, after only 3, 6, and 9 min. No *S. enterica* survivors were detected in that same juice after 12 and 15 min (Figure 1A). Irrespective of plating medium, lemon juice with added yucca extract and thyme oil (0.2%) exhibited the strongest bactericidal effect against the pathogen (Figures 1A,B). For those two juices that contained thyme oil (0.1 and 0.2%), higher numbers of *S. enterica* survivors were observed on TAL medium compared to XLT-4 agar (*P* < 0.05).

### Survival of *Salmonella* on Raw Chicken Meat

**Figure 2:**
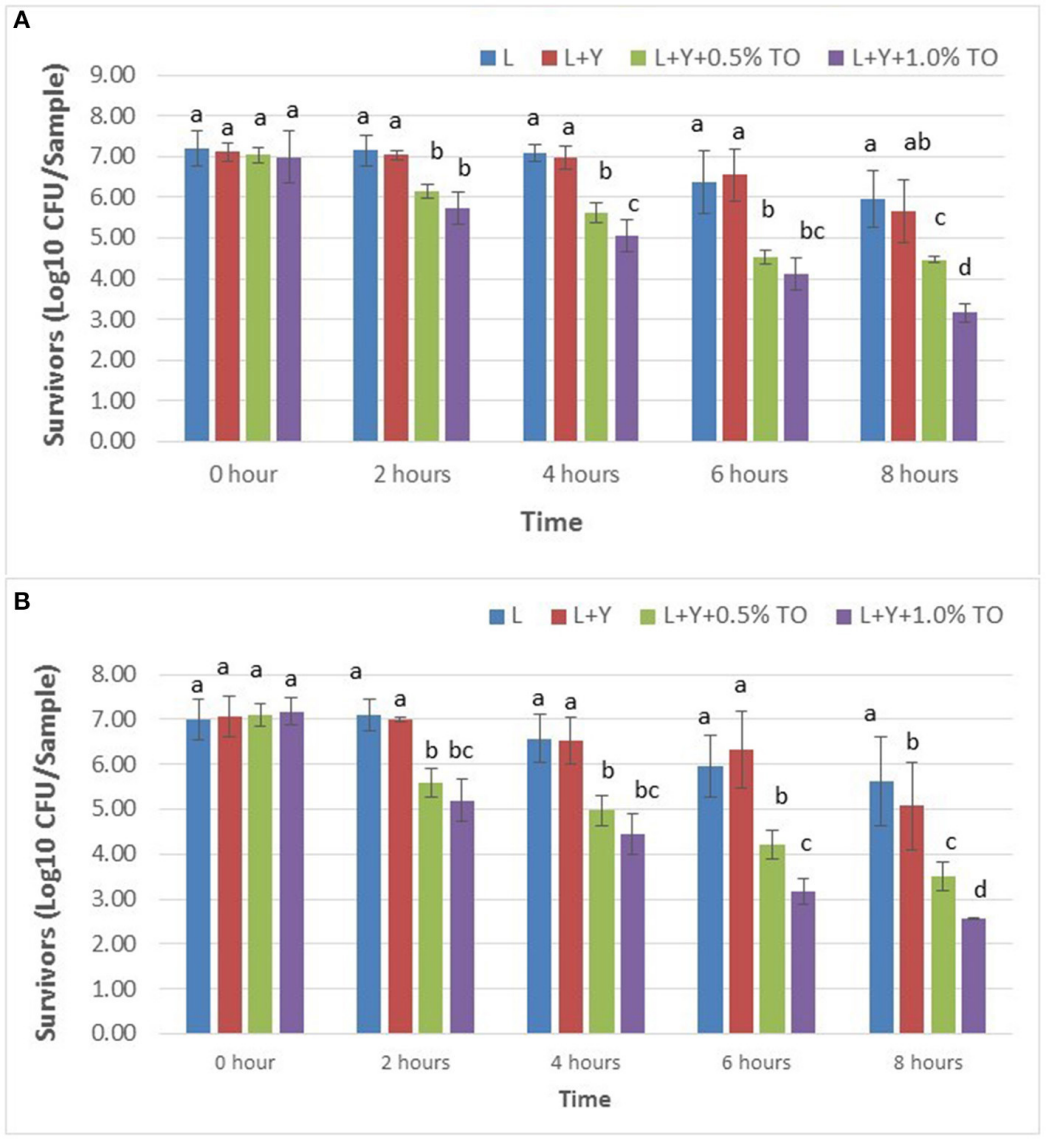
Survivors of *Salmonella enterica* attached to raw chicken breast meat in lemon juice marinades based on bacterial colony counts on TAL medium **(A)** and on XLT4 agar **(B)**.

No salmonellae was detected on non-inoculated samples of chicken breast meat. The numbers of *S. enterica* survivors on marinated raw chicken breast based on colony counts on TAL medium and XLT4 agar, respectively, are presented in Figures 2A,B. The initial viable count of *S. enterica* on artificially inoculated chicken breast filets was ~8.0 Log_10_ CFU/sample based on microbial analysis of the cell suspension used to inoculate the chicken samples. The numbers of survivors on the non-treated inoculated chicken breast after 2 h at ambient temperature (22 ± 1°C) were ~7.08 log CFU/sample representing a 0.92 Log CFU decrease in cell viability. Compared to lemon juice alone or juice with added yucca extract, all treatments containing thyme oil significantly reduced initial numbers of viable *S. enterica* on raw chicken irrespective of the type of agar medium used for counting bacterial colonies (*P* < 0.05). After 8 h, initial numbers of viable *S. enterica* on chicken in lemon juice alone and with yucca extract decreased from 7.08 to 5.96 and 5.66 Log CFU/sample, respectively, based on numbers of survivors on TAL medium (Figure 2A). In contrast, after 8 h, initial numbers of the pathogen (Log CFU/sample) on chicken in marinades with added thyme oil decreased to 4.46 (0.5% TO) and 3.17 (1.0% TO) (Figure 2A). Higher numbers of *Salmonella* survivors were consistently recovered on TAL medium compared to XLT agar.

### Decimal Reduction Times (D-Values)

Table 1 shows the effect of marination on D-values for *S. enterica* planktonic cells (A) and cells attached to raw chicken breast meat (B). No significant differences in D-values were observed for cells treated with lemon juice alone compared to lemon juice containing only yucca extract irrespective the state of the cells (planktonic or attached) or plating medium (*P* > 0.05). For planktonic cells in marinade solutions the addition of thyme oil (0.1 or 0.2%) significantly decreased the D-value of the pathogen compared to control (lemon juice and lemon juice + yucca extract) irrespective of the plating medium (*P* < 0.05). Based on numbers of pathogen survivors on TAL medium, a similar observation was made for cells attached to chicken whereby significant reductions in D-values (*P* < 0.05) occurred with the addition of thyme oil to marinade solutions (Table 1B). Based on numbers of survivors on XLT-4 agar, no significant differences in D-values (*P* > 0.05) were observed for *S. enterica* cells attached to marinated raw chicken (Table 1B).

**Table 1:** Decimal reduction times (D values) for *Salmonella enterica* as planktonic cells **(A)** and cells attached to raw chicken breast meat **(B)** during exposure to lemon juice marinade solutions at 22 ± 1°C.

	**D value (minutes)^x^**	
**Treatment**	**TAL**	**XLT4**
**(A) PLANKTONIC CELLS**		
Lemon juice	4.83 ± 0.18^a^	3.55 ± 0.26^a^
Lemon juice + yucca^*^	5.21 ± 0.44^a^	3.50 ± 0.08^a^
Lemon juice + yucca + 0.1% thyme oil	2.99 ± 0.21^b^	2.41 ± 0.40^b^
Lemon juice + yucca + 0.2% thyme oil	1.02 ± 0.46^c^	0.74 ± 0.28^c^
	**D value (hours)^x^**	
**Treatment**	**TAL**	**XLT4**
**(B) ATTACHED CELLS**		
Lemon juice	4.59 ± 1.39^a,b^	4.04 ± 1.50^a^
Lemon juice + yucca	6.44 ± 2.09^a^	3.68 ± 1.54^a^
Lemon juice + yucca + 0.5% thyme oil	2.46 ± 0.19^b^	2.04 ± 0.15^a^
Lemon juice + yucca + 1.0% thyme oil	2.25 ± 0.33^b^	1.84 ± 0.22^a^

* *yucca extract (0.5%); TAL, thin agar layer medium; XLT4, xylose lysine tergitol agar*.

x *For each group of cells (planktonic or attached) average D values with different superscripts (a, b) within a column are significantly different (P < 0.05)*.

### Sub-lethal Injury in *Salmonella* Survivors

Table 2 shows sub-lethal injury (expressed by linear regression parameters) in *S. enterica* survivors resulting from exposure of inoculated chicken breast meat to the marinade solutions. That table displays the slopes and intercepts from linear regression plots, which showed reduction in culturability of the pathogen. Based on those slope and intercept parameters, the extent of sub-lethal injury in pathogen survivors caused by each of the marinade treatments was not statistically significant (*P* > 0.05).

**Table 2:** Slopes and intercepts from regression plots for reduction in viability of *Salmonella enterica* on chicken breast meat in lemon juice marinade.

**Treatment**	**^a^Slope**	**^b^Intercept**	**R-Squared**
Lemon juice alone	1.283 ± 0.227	0.349 ± 0.227	0.918
Lemon+ Yucca	1.223 ± 0.066	0.104 ± 0.047	0.857
Lemon+ Yucca+ 0.5% Thyme oil	1.235 ± 0.245	−0.222 ± 0.736	0.907
Lemon+ Yucca+ 1.0% Thyme oil	1.204 ± 0.108	0.431 ± 0.291	0.889

a *Values for slope are not significantly different from 1.0 (P > 0.05)*.

b *Values for intercept are not significantly different from 0 (P > 0.05)*.

*Each value represents the mean ± standard deviation from 3 replicate experiments*.

### The pH of Marinade Solutions

The pH value of the lemon juice was 2.44 and its citric acid content was 7.0 g/100 mL. The pH values for marinade solutions (22 ± 1°C) with or without raw chicken are shown in Table 3. The initial pH of the marinades ranged from 2.44 to 2.46. The pH of control marinade solution (without raw chicken) remained largely unchanged (pH ~ 2.44) through 8 h. In contrast, significant increases (*P* < 0.05) in the pH values were observed for all marinades that contained raw chicken. Increases in pH values at 4 and 8 h averaged 0.16 and 0.44, respectively. Compared to control, all other marinades exhibited a higher pH value after 2, 4, 6, and 8 h in contact with raw chicken samples (*P* < 0.05) with pH values ranging from 2.88 to 2.90 after 8 h.

**Table 3:** Changes in pH of marinade solutions during marination of non-inoculated raw chicken breast meat at 22 ± 1°C.

	**^a^pH of marinade solutions**				
**Treatment**	**0 h**	**2 h**	**4 h**	**6 h**	**8 h**
L (NC)^*^	2.44 ± 0.01Ax	2.44 ± 0.02Ay	2.43 ± 0.05Ay	2.45 ± 0.02Ay	2.44 ± 0.02Ay
L	2.44 ± 0.02Ex	2.51 ± 0.03Dx	2.59 ± 0.04Cx	2.66 ± 0.02Bx	2.89 ± 0.02Ax
L + Y	2.46 ± 0.02Ex	2.54 ± 0.04Dx	2.62 ± 0.03Cx	2.68 ± 0.01Bx	2.88 ± 0.02Ax
L + Y + 0.5% TO	2.45 ± 0.03Ex	2.55 ± 0.02Dx	2.60 ± 0.02Cx	2.68 ± 0.02Bx	2.90 ± 0.02Ax
L + Y + 1.0% TO	2.46 ± 0.02Dx	2.54 ± 0.04Cx	2.64 ± 0.02Bx	2.67 ± 0.03Bx	2.88 ± 0.03Ax

a *Each pH value represents the mean ± standard deviations of three replicate experiments*.

* *NC, no chicken in marinade*.

*L, lemon juice; Y, yucca extract (0.5%); TO, thyme oil*.

*Means that do not share the same letter (A, B, C, D, E) within the same row or within each column (x, y) are significantly different (P < 0.05)*.

### GC-MS Analysis of Thyme Oil

Results of gas chromatography-mass spectrometry (GC-MS) analysis of thyme oil used in the present study are presented in Table 4. Fifteen different components representing major and minor components of that essential oil were identified at concentrations ranging from 0.14 to 51.07%. Thymol and O-cymene were the top two major components at concentrations of 51.07 and 24.1%, respectively.

**Table 4:** Compounds identified in thyme oil based on analysis using GC-MS.

**Component**	**RT**	**RI_**s**_**	**RI_**Nist**_**	**Percent (%)**
α-pinene	5.98	942.5	937	1.78
Camphene	6.37	956.8	952	0.42
β-Myrcene	7.29	990	991	0.55
**o-Cymene**	8.24	1,024.3	1,022	**24.1**
Eucalyptol	8.45	1,032	1,032	1.67
γ-Terpinene	9.14	1,057.2	1,060	5.42
4-Carene/Linalool	10.27	1,097.9	1,099	4.4
Camphor	11.63	1,147	1,145	1.49
Isoborneol	12.09	1,164	1,157	0.14
Endo-borneol	12.32	1,172.6	1,167	0.73
Terpinen-4-ol	12.56	1,181.2	1,177	0.72
**Thymol**	15.57	1,293.2	1,291	**51.07**
Carvacrol	15.82	1,301.5	1,299	3.33
Caryophyllene	18.99	1,422.6	1,419	0.2
Caryophyllene oxide	22.95	1,585.4	1,581	0.18

*RT, Retention time; RI_s_, Kovat′s Retention index of thyme oil compounds found in the sample used in this study; RI_Nist_, Kovat′s Retention index in NIST14 library. Values in bold type indicate the amounts (%) of two major components of thyme oil*.

## Discussion

*In vitro* microbial susceptibility tests such as MIC and MBC, are usually performed to evaluate the sensitivity of an organism to an antimicrobial agent such as an antibiotic or chemical preservative. Since the MIC and MBC of thyme oil depend on several variables including composition and concentration of this EO's bioactive components and the cultural conditions for the test organisms, comparison of results with those of other studies involving thyme oil is not simple (39). Considering these limitations, Lu and Wu (40) reported 0.1 and 0.2% as MIC and MBC, respectively, for each of four *S. enterica* serotypes namely, Typhimurium, Enteritidis, Sefentenberg, and Kentucky. Those concentrations are higher than those (MIC = 0.03% and MBC = 0.06%) for the five serovar mixture (Enteritidis ATCC13076, Heidelberg ATCC 8326, Typhimurium ATCC 14802, Gaminara ATCC 8324, and Oranienburg ATCC 9239) reported in the present study. While the MIC and MBC concentrations differ between the two studies, one common finding is that the MBC is twice that of the MIC. In this respect, when the ratio of MBC to MIC is ≤ 4, the antimicrobial is bactericidal (41). Since the MBC:MIC ratio of thyme oil for *S. enterica* is 2, that EO is bactericidal and should be effective for augmenting the antibacterial activity of citrus juice marinades against *S. enterica*.

Citrus juices from lime and lemon fruit are major sources of citric acid and are widely used by consumers to marinate raw poultry meat in preparation for cooking. Several published reports have highlighted consumers' belief that diluted lemon juice, lime juice, or vinegar may destroy pathogens on raw poultry meat to improve microbial safety of this product (42, 43). In the present study, exposure of planktonic *S. enterica* cells to undiluted lemon juice (pH 2.44) at 22 ± 1°C for 15 min decreased initial numbers of the pathogen by 3.7 Log CFU/mL based on numbers of survivors on TAL medium (Figure 1A). This result indicates that lemon juice alone exerts a bactericidal effect on *S. enterica*. The low pH (2.44) of the lemon juice plus the citric acid in that juice are likely responsible for the observed antimicrobial action as similar findings were reported by others (22, 44). The 0.92 Log CFU decrease in initial numbers of *S. enterica* after 2 h of inoculating the chicken suggests a loss of viability of some cells during drying of the inoculum at ambient temperature (22 ± 1°C). In this regard, 7.08 Log CFU/sample was used as the actual initial viable count for calculating reductions in populations of the pathogen on chicken breast meat.

When samples of inoculated raw chicken breast meat were marinated for 8 h in lemon juice alone, reduction in the initial viable count was only 1.12 Log CFU/sample (Figure 2A) which was lower than that observed for planktonic cells exposed to lemon juice for 15 min (Figure 1A). These results are not surprising considering the fact that bacteria attached to surfaces are more tolerant to antimicrobial agents compared to planktonic bacteria (45). For example, *Salmonella* cells attached to surfaces exhibited far more resistance than planktonic cells to disinfectants used during poultry processing (46). Moreover, the increased resistance of the attached cells may be partly attributed to the protective effect that the chicken breast offers *S. enterica*. It is likely that cell attachment to the chicken meat surface precludes full contact of the pathogen with the marinade treatment solutions. Additionally, production of biofilm on the meat surface could offer some protection to the attached cells. Dimakopoulou-Papazoglou et al. (47) reported an increased tendency of *S. enterica* to produce biofilm rapidly especially when they were exposed to conditions of very low pH. However, for planktonic *S. enterica* cells, such protection via attachment and biofilms is not possible because the cells are directly exposed to marinade solutions.

The survival of *S.enterica* on chicken breast meat in lemon juice suggests that, depending on the initial level of *Salmonella* contamination, survivors of that pathogen may persist on raw chicken in lemon juice even after several hours and pose a health risk to consumers. Our results are supported by those of Yang et al. (22) who reported that lemon juice marinade (pH 2.6–2.8) was not effective for completely inactivating *Salmonella* Enteritidis, *Escherichia coli* O157:H7, and *Listeria monocytogenes* on raw beef. In the present study, the addition of thyme oil (0.1 or 0.2% wt/vol) to the lemon juice significantly (*P* < 0.05) increased in the bactericidal effect of lemon juice marinades against both planktonic (Figure 1A) and attached cells (Figure 2A) of *S. enterica*.

There are several published reports on the potential of essential oils (EOs) including thyme oil to control foodborne pathogens due to their bacteriostatic and bactericidal properties (24, 26, 27, 29, 48–50). Although the precise antibacterial mode of action of thyme oil is not fully elucidated, the antimicrobial effects of this EO might be attributed to additive or synergistic effects of several of its major and/or minor components (26, 51). The results of GC-MS analysis of the composition of the thyme oil used in the present study (Table 4) revealed that thymol (51.1%) and O-cymene (24.1%) were two major components. Recently, compositional analysis of thyme oil extracted from thyme leaves in Cordoba, Argentina, revealed that the two major components of that EO were also thymol and O-cymene at concentrations of 34.8 and 37.1%, respectively (52). (53) reported that the antibacterial mode of action of thymol involves increased cell permeability, dissipation of the pH gradient across the cytoplasmic membrane, and cellular leakage of inorganic ions. The other components of thyme oil may further contribute to the bactericidal properties as they may act synergistically in contribution to thyme oil's antibacterial effect. For example, p-cymene, another main component of essential oils, has relatively weak antimicrobial activity; however, it may enhance the antimicrobial action of other EO components via synergism and additive effects ((54))

Other plausible factors that likely contributed to the antibacterial effect of lemon juice marinade containing thyme oil are increased hydrophobicity and miscibility of the thyme oil. Hydrophobicity of EOs increases at low pH levels (55) and increased hydrophobicity enhances partitioning of EOs in bacterial membrane lipids to disrupt cell functioning (26, 56, 57). In this regard, lemon juice (pH 2.44) used in the present study, served as a low pH medium for enhancing the bactericidal effect of thyme oil against *Salmonella* on raw chicken meat. The hydrophobic characteristic of thyme oil precludes its miscibility in the lemon juice, which is hydrophilic. Poor miscibility EOs in water-based solutions is one of the challenges to their widespread application in foods systems (29, 30). When an antimicrobial agent is poorly miscible in foods, it does not readily contact foodborne microorganisms to exert its bacteriostatic or bactericidal effect. Improved miscibility of EOs via use of emulsifiers can enhance their antimicrobial activity (58). To improve the miscibility of EOs, researchers have used synthetic emulsifiers such as Tween 20 or Tween 80 (24, 59, 60). However, considering growing consumer demand for more natural alternatives to synthetic food additives, we used yucca extract in the present study to emulsify the thyme oil in the lemon juice. Thomas-Popo et al. (31) were the first researchers to report the application of yucca extract for solubilizing an EO component (isoeugenol) in raw pineapple juice to kill enteric pathogens. Yucca extract from the Mohave Yucca plant (*Yucca schidigera)* is a natural surfactant, which is FDA-approved for use in the food, cosmetic, and feed industries (61). That plant extract contains saponins, which have both lipophilic and hydrophilic characteristics (62). Also, saponins are known to have antimicrobial properties (63). Based on the previously stated information, we speculate that both the low pH of the lemon juice (pH 2.44) and the emulsifying property of yucca extract improved the antibacterial activity of thyme oil against *S. enterica* as planktonic cells and as cells attached to chicken breast meat.

The D-values (Table 1) for *S. enterica* exposed to marinade solutions represent the times required for a 10-fold (1.0 Log) destruction of the initial viable population of the pathogen. The significant (*P* < 0.05) decreases in D-value for planktonic cells of *S. enterica* in marinades containing thyme oil at 0.1 and 0.2% (v/v) suggest a faster death rate of the pathogen in those marinades compared to lemon juice alone or with added yucca extract (Table 1A). Based on numbers of survivors on TAL medium, the death rate for *S. enterica* cells attached to chicken breast meat was significantly (*P* < 0.05) faster in lemon juice marinades with added thyme oil at 0.5 and 1.0% (v/v) compared to lemon juice with added yucca extract (Table 1B). Most of the antimicrobial activity of thyme oil seem to be associated with its phenolic components such as carvacrol and thymol (64–66). The antimicrobial action of phenolic compounds is mainly associated with membrane disruption in Gram-negative and Gram-positive bacteria (67). While carvacrol constitutes only 3.33% of the components of thyme oil used in the present study, thymol (51%) is a major phenolic component (Table 4). The increased death rate of *S. enterica* cells in lemon juice with added thyme oil further suggests that the antimicrobial components of thyme oil inflicted additional damage to the pathogen beyond that caused by the low pH of the lemon juice.

Our observed increase in pH of the marinades (22 ± 1°C) during 8 h of marination of chicken breast meat (Table 3) was likely due to dilution of the marinade by juices from the raw chicken. Similar increases in pH of meat marinades have been reported (12, 68). Tan et al. (69) demonstrated that raw chicken meat immersed in a buffered saline solution (pH 2.0) at 4°C increased the pH of that medium to 4.74 after 24 h. In those previously mentioned reports, the increases in pH were likely attributed to the high buffering capacity of meat proteins (12). The buffering effect of chicken meat protected *S. enterica* from the effects of acidic pH (69). Our findings and those of others regarding the increase in pH of acidic solutions containing poultry meat suggest that raw poultry meat can decrease the inhibitory properties of acidic marinades over time. This in turn can reduce the lethal effect of the marinade and allow survival of human enteric pathogens to pose a food safety risk to consumers. This problem is further exacerbated considering that some *Salmonella* serovars possess acid-adaptation systems that enhance their survival at pH levels as low as 2.5 (70, 71). After 8 h, in spite of the increase in pH of the marinades containing raw chicken breast meat, the added thyme oil (1.0% v/v) inactivated the initial population of *S. enterica* by 3.91 and 4.52 Log CFU/sample, based on survivors on TAL medium and XLT4 agar, respectively (Table 2) (*P* < 0.05). Based on this result thyme oil at 1.0% (v/v) exhibits good potential for killing *S. enterica* on chicken meat in acidic marinades that undergo increases in pH during several hours of marination at ambient temperature (22 ± 1°C).

Despite recommendations by government agencies globally (72–74) that meats should be marinated under refrigeration, some consumers marinate meats at room temperature. This is especially true in some areas of the world where refrigeration is unavailable. In the present study, raw chicken breast meat immersed in lemon juice was held at 22 ± 1°C for 8 h to simulate temperature abuse during marination. The recommendation for marinating meats under refrigeration (<5°C) for no more than 2 days (74) is to prevent growth of pathogenic microorganisms. In the present study, addition of thyme oil to lemon juice marinade significantly increased the death rate of *S. enterica* on the chicken compared to marinade without thyme oil (*P* < 0.05). These findings suggest that thyme oil can decrease the survival of *S. enterica* in lemon juice marinade under temperature abuse conditions for 8 h. Therefore, addition of thyme oil to acidic marinades may serve as an alternative to refrigeration to ensure microbial safety of chicken breast meat for 8 h at room temperature.

In determining the numbers of *S. enterica* survivors in lemon juice marinades, we plated diluted samples of marinade on two agar media, namely, TAL medium and XLT4 agar. Two agar media were used to evaluate the extent of sub-lethal injury in *S. enterica* survivors. Hurst (75) described sub-lethal injury as a result of exposure of microbes to a chemical or physical process that damages but does not kill them. The TAL medium allowed resuscitation and growth of sub-lethally injured survivors and growth of non-injured survivors of the pathogen without interference (growth) from background organisms (35, 76). The XLT4 agar is a selective medium, which allowed growth of non-injured survivors while preventing growth of sub-lethally injured organisms and background microflora. Therefore, the occurrence of larger populations of *S. enterica* on TAL medium compared to XLT4 agar indicated sub-lethal injury. For both planktonic cells and cells attached to raw chicken, numbers of *S. enterica* survivors were significantly higher on TAL medium compared to XLT4 agar (*P* < 0.05). On this basis, these results indicate that the acidic marinade treatments caused sub-lethal injury in the part of the surviving population of *S. enterica*. However, based on the slope intercept method described by Wuytack et al. (36) the extent of sub-lethal injury caused by each of the marinades was not statistically significant (*P* > 0.05; Table 3).

The consistently lower numbers of *S. enterica* survivors observed on XLT4 agar suggests that the use of selective media for evaluating pathogen survivors in acidic marinades can erroneously overestimate the extent of inactivation of the target pathogen. This is because selective media such as XLT4 are unable to support growth of injured pathogens (77). More importantly sub-lethally injured organisms might be able to resuscitate and develop increased resistance to antimicrobial treatments (78). From a food safety perspective, our results are important considering that some *S. enterica* serovars have a low infectious dose (79). Therefore, failure to detect even small amounts of that pathogen due to sub-lethal injury causes critical limitations in food diagnostics because of overestimating pathogen inactivation and the possibility of false negative results (80).

## Conclusions

*Salmonella enterica* can persist for several hours on raw chicken breast meat during marination in lemon juice at room temperature (22°C). The addition of thyme oil at 0.5 or 1.0 % (v/v) to lemon juice marinade containing yucca extract significantly increases the death rate of *S. enterica* on raw chicken during marination at 22°C. Thyme oil combined with yucca extract can improve the antimicrobial effectiveness of lemon juice against *Salmonella* on raw chicken breast and enhance the microbial safety of this popular poultry product.

## Data Availability Statement

The raw data supporting the conclusions of this article will be made available by the authors, without undue reservation.

## Author Contributions

AM conceived the research idea, designed the study, and guided the research performed by SK. SK performed the experiments and drafted the manuscript. RM performed GC-MS analysis of thyme oil. ET-P and SK performed statistical analysis of the data. JD, AS, ET-P, SW, and SI provided revisions of the manuscript. All authors contributed to the article and approved the submitted version.

## Conflict of Interest

The authors declare that the research was conducted in the absence of any commercial or financial relationships that could be construed as a potential conflict of interest.
